# Soret and Dufour effects on unsteady mixed convection slip flow of Casson fluid over a nonlinearly stretching sheet with convective boundary condition

**DOI:** 10.1038/s41598-017-01205-5

**Published:** 2017-04-25

**Authors:** Imran Ullah, Ilyas Khan, Sharidan Shafie

**Affiliations:** 1 Department of Mathematical Sciences, Faculty of Science, Universiti Teknologi Malaysia, 81310 UTM Johor Bahru, Johor, Malaysia; 2 Basic Sciences Department, College of Engineering Majmaah University, Majmaah, 11952 Saudi Arabia

## Abstract

Unsteady mixed convection flow of Casson fluid towards a nonlinearly stretching sheet with the slip and convective boundary conditions is analyzed in this work. The effects of Soret Dufour, viscous dissipation and heat generation/absorption are also investigated. After using some suitable transformations, the unsteady nonlinear problem is solved by using Keller-box method. Numerical solutions for wall shear stress and high temperature transfer rate are calculated and compared with previously published work, an excellent arrangement is followed. It is noticed that fluid velocity reduces for both local unsteadiness and Casson parameters. It is likewise noticed that the influence of a Dufour number of dimensionless temperature is more prominent as compared to species concentration. Furthermore, the temperature was found to be increased in the case of nonlinear thermal radiation.

## Introduction

The study of boundary layer flow over stretching sheets has attracted attention of several investigators due to its enormous applications in many engineering processes such as extrusion of polymer sheet from dye, cooling of metallic sheets, paper production, glass fiber, enhanced recovery of petroleum resources. In the manufacturing process of polymers, the desired characteristics of the end product highly depend on stretching rate, cooling rate and stretching process. During preparation of these sheets, the desired thickness can be achieved by stretching the sheet as melt exude from slit. Knowing its applications, Crane^1^ for the first time explored the boundary layer flow caused by linearly stretching sheet. Later on, Gupta and Gupta^2^ extended this problem to porous stretching surface and pointed out that sheet velocity does not oblige to be linear. This opened new doors for investigators to further explore the stretching sheet problem. Ali^3^ took the initiative and analyzed boundary layer flow of viscous fluid over nonlinearly stretching sheet. Inspired by this, Cortell^4^ extended the work of Ali^3^ and considered two cases of temperature viz. constant surface temperature and prescribed surface temperature along the sheet. Kechil and Hashim^5^ further explored the effect of chemical reaction on a viscous fluid past nonlinearly stretching sheet through a porous medium.

Moreover, Hayat *et al*.^6^ investigated electrically conducting flow of micropolar fluid due to nonlinearly stretching sheet. Hsiao^7^ discussed the effects of thermal radiation on incompressible flow of viscous fluid caused by nonlinearly stretching sheet. The influence of dissipating heat on heat transfer flow past nonlinearly stretching sheet is reported by Alinejad and Samarbukhsh^8^. Anwar *et al*.^9^ analyzed the radiation effects on electrically conducting flow of nanofluid over nonlinearly stretching sheet.

Aforementioned studies are restricted to steady flow caused by stretching a sheet, however in some cases, the flow field and heat transfer may be unsteady owing to sudden stretching of the sheet or by step change of sheet temperature. The unsteadiness is because of change in wall velocity or wall temperature etc. Nazar *et al*.^10^ examined two dimensional unsteady stagnation point flow anticipated to stretching sheet and obtained similarity solutions by Keller-box method. Elbashbeshy and Bazid^11^ obtained the similarity solutions for unsteady flow of viscous fluid towards stretching sheet with heat transfer. Sharidan *et al*.^12^ investigated the characteristic features of variable heat flux in unsteady boundary layer flow over stretching surface using similarity transformations. Ishak *et al*.^13^ studied unsteady mixed convection flow of a Newtonian fluid past linearly stretching sheet. The effects of suction/injection on unsteady boundary layer flow of Newtonian fluid due to stretching sheet in the presence of chemical reaction have been put in place by Chamkha *et al*.^14^. In another study, Freidoonimehr *et al*.^15^ explored hydromagnetic unsteady viscous flow of nanofluid towards a porous stretching surface.

It is generally known that the complex nature of non-Newtonian fluids offers a challenge to modelers, engineers, mathematicians and physicists. The governing equations appear in these fluids, are strongly nonlinear and are much complicated. In order to insight a clear picture of non-Newtonian fluids and their diverse applications, it is important in order to further investigate their flow behavior. It is also a widespread belief that there is not a single equation exists which demonstrates all characteristics of non-Newtonian fluids. Several constitutive models have been made to clarify the behavior of non-Newtonian fluids. Among them, Casson fluid is an important non-Newtonian fluid that exhibit yield stress. Casson fluid is defined as shear thinning fluid which is assumed to have an infinite viscosity at zero shear rate. Further, Casson model is also preferable model for rheological data in comparison to viscoplastic models. Moreover, Human blood is also of the view as Casson fluid. Mukhopadhyay^16^ examined two dimensional unsteady flow of Casson fluid due to linearly stretching sheet. The impact of thermal radiation on Casson fluid towards nonlinearly stretching the sheet in the presence of heat source/sink is investigated by Sumalatha and Bandari^17^.

Besides, the flow owing to steady or unsteady stretching sheet, the effect of mixed convection caused by buoyancy force could not be ignored for the stretching sheet. There has been growing interest in analyzing the problem of thermal radiation with mixed convection boundary layer flow because of its important applications in space technology, geothermal engineering and cooling of nuclear reactors. Two dimensional unsteady mixed convection flow over a permeable stretching sheet in the presence of thermal radiation is reported by Mukhopadhyay^18^. Hsiao^19^ also investigated the influence of thermal radiation on mixed convection flow induced by stretching surface. Further, Pal and Mondal^20^ analyzed the effects of chemical reaction on mixed convection flow of viscous fluid caused by nonlinearly stretching sheet integrated into a porous medium in the presence of thermal radiation. Motivated by this, Makinde^21^ examined the characteristics of heat and mass transfer on mixed convection flow of Newtonian fluid past a stretching surface in the presence of thermal radiation.

On the other hand, a phenomenon of thermo-diffusion in liquids was presented by Soret in 1879, and observed that concentration gradient is a happen cause of temperature gradient in a direct diffusion. A reciprocal effect, in which a difference of temperature is caused by the gradient of species concentration was developed by Dufour in 1872. In several applications, the effect of Soret and Dufour is often neglected, since the order of magnitude is smaller than the effect described by Fourier’s and Fick’s laws. However, both effects become significant when the species having lower density than the surrounding liquids are introduced at surface of fluid medium. Also, such effects play a vital role in the field of geosciences, oceanography, chemical engineering and air pollution. Keeping in view of its important applications, Hayat *et al*.^22^ investigated the effects of Soret and Dufour on two dimensional flow of Casson fluid generated due to stretching surface in the presence of magnetic field. The thermophoretic and Soret Dufour effect on steady flow past a non-isothermal wedge in the presence of heat generation and nonlinear thermal radiation was analyzed numerically by Pal and Mondal^23^. Khan *et al*.^24^ studied the Soret Dufour effect on electrically conducting flow of viscous fluid in the presence of first order chemical reaction. Recently, Pal *et al*.^25^ successfully discussed the influence of Soret and Dufour on heat and mass transfer flow of Newtonian fluid over nonlinearly stretching sheet submerged in a nanofluid in the presence of linear thermal radiation.

All the above studies dealt without slip condition at the wall. Nevertheless, in certain physical situations the momentum slip at wall is unavoidable. Especially, for some non-Newtonian fluids no slip condition at wall is in sufficient. For example, melting polymer always possesses velocity slip at wall. Applications of fluids having slip condition at wall include polishing of artificial heart valves and internal cavities. Bhattacharyya and Layek^26^ considered the velocity slip at wall and analyzed boundary layer flow of viscous fluid induced by porous stretching sheet in the presence of chemical reaction. Motivated by this, Bhattacharyya *et al*.^27^ studied the mechanism of slip condition on unsteady stagnation point flow due to stretching sheet. The hydrodynamic slip effects on Casson fluid towards permeable stretching sheet in the presence of thermal radiation and chemical reaction was reported by Poornima *et al*.^28^. Recently, Shen *et al*.^29^ explored the influence of slip condition at wall and prescribed heat flux on electrically conducting mixed convection flow of Newtonian fluid due to nonlinearly stretching surface.

Another important mechanism in the study of boundary layer flow of Newtonian and non-Newtonian fluids is the convective boundary condition at the boundary wall. A bulk of literature is devoted to constant wall temperature and constant heat flux at the wall. However, consideration of convective heat transfer at temperature wall is more practical. Makinde and Aziz^30^ analyzed numerically boundary layer flow of nanofluid induced by stretching sheet with a convective boundary condition and concluded that the Biot number enhances thickness of thermal boundary layer significantly. Later, Ishak *et al*.^31^ explored the effects of thermal radiation on boundary layer flow of viscous fluid past a moving plate with a convective boundary condition. On the other hand, Nadeem *et al*.^32^ and Mahanta and Shaw^33^ investigated the mechanism of convective boundary condition for three dimensional hydromagnetic flow of Casson nanofluid and Casson fluid caused by linearly and nonlinearly stretching sheet, respectively. Very recently, Oyelakin *et al*.^34^ discussed the heat and mass transfer characteristics in unsteady Casson nanofluid induced by stretching a sheet with a convective boundary condition.

From above discussion, it is very much clear that Soret Dufour effect on unsteady mixed convection flow of non-Newtonian Casson fluid due to nonlinear stretching sheet has not yet been reported. Very less attention is paid towards the heat and mass transfer flow under the influence of nonlinear thermal radiation and thermophoresis. The objective of the present analysis is to explore the mechanisms of slip and convective boundary conditions on unsteady electrically conducting flow of Casson fluid induced by nonlinearly stretching sheet under the influence of Soret and Dufour magnetic field. The governing equations are solved numerically by Keller-box method^35^ taking advantage of similarity transformations. Physical interpretations for velocity, temperature, velocity gradient and temperature gradient are achieved through graphs.

## Mathematical Formulation

**Figure 1:**
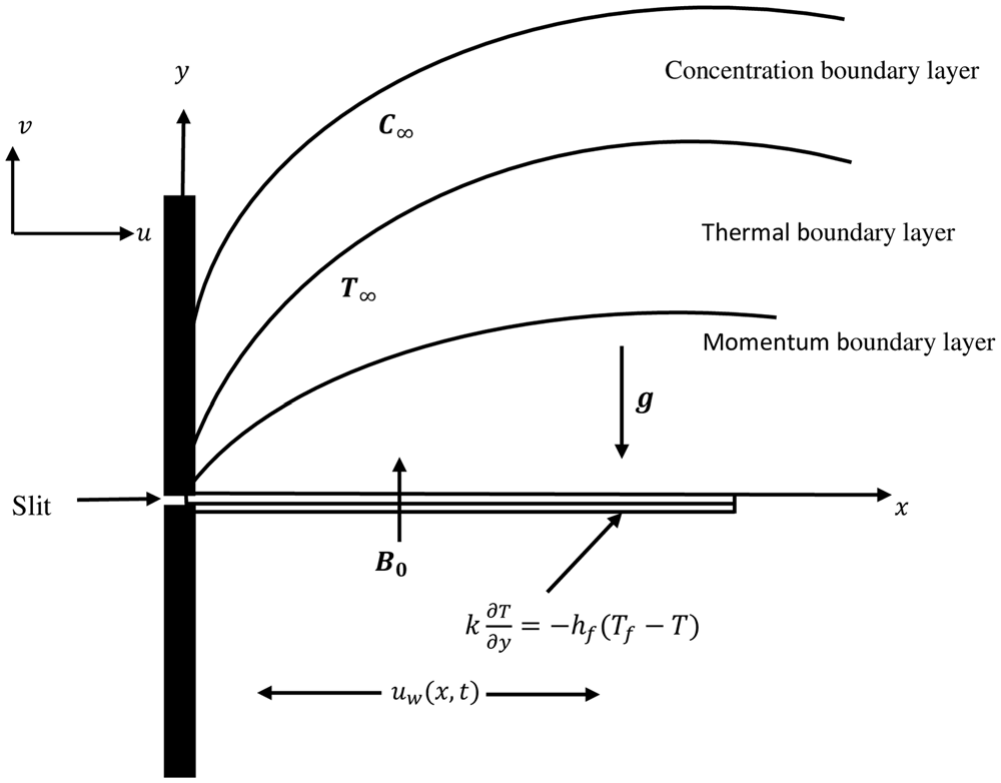
Physical model and coordinate system.

The unsteady two dimensional mixed convection flow of Casson fluid due to nonlinearly stretching sheet in the presence of nonlinear thermal radiation is considered. Further, the momentum slip is also taken into account at wall. It is assumed that sheet is stretched with the velocity ${u}_{w}(x,t)=a{x}^{n}/(1-\gamma t)$ and the free stream velocity is ${u}_{e}(x,t)=b{x}^{n}/(1-\gamma t)$ where *a*, *b* and *γ* are constants and *t* is time (see Fig. 1). A transverse magnetic field is applied perpendicular to the stretching sheet. The induced magnetic field is neglected due to small enough magnetic Reynolds number. Further, the wall of sheet is heated by fluid with temperature *T*_*f*_ and free stream temperature is denoted by *T*_∞_. Moreover, species concentration at wall is denoted by *C*_*w*_ and away from the wall is represented by *C*_∞_.

The rheological governing equations for momentum, energy and concentration are given as:

1 $$\frac{\partial u}{\partial x}+\frac{\partial \upsilon }{\partial y}=0,$$

2 $$\begin{array}{rcl}\frac{\partial u}{\partial t}+u\frac{\partial u}{\partial x}+\upsilon \frac{\partial u}{\partial y} & = & \frac{\partial {u}_{e}}{\partial t}+{u}_{e}\frac{\partial {u}_{e}}{\partial x}+\nu (1+\frac{1}{\beta })\frac{{\partial }^{2}u}{\partial {y}^{2}}+\frac{\sigma {B}^{2}(x,t)}{\rho }\\  &  & \times ({u}_{e}-u)+g{\beta }_{T}(T-{T}_{\infty })+g{\beta }_{C}(C-{C}_{\infty }),\end{array}$$

3 $$\begin{array}{rcl}\frac{\partial T}{\partial t}+u\frac{\partial T}{\partial x}+\upsilon \frac{\partial T}{\partial y} & = & \alpha \frac{{\partial }^{2}T}{\partial {y}^{2}}-\frac{1}{\rho {c}_{p}}\frac{\partial {q}_{r}}{\partial y}+\frac{\nu }{{c}_{p}}(1+\frac{1}{\beta }){(\frac{\partial u}{\partial y})}^{2}+\frac{\sigma {B}^{2}(x,t)}{\rho {c}_{p}}\\  &  & \times {({u}_{e}-u)}^{2}+\frac{Q(x,t)}{\rho {c}_{p}}(T-{T}_{\infty })+\frac{D{}_{m}k_{T}}{{c}_{s}{c}_{p}}\frac{{\partial }^{2}C}{\partial {y}^{2}},\end{array}$$

4 $$\frac{\partial C}{\partial t}+u\frac{\partial C}{\partial x}+\upsilon \frac{\partial C}{\partial y}=D\frac{{\partial }^{2}T}{\partial {y}^{2}}-\frac{\partial }{\partial y}{V}_{T}(C-{C}_{\infty })+\frac{D{}_{m}k_{T}}{{c}_{s}{c}_{p}}\frac{{\partial }^{2}T}{\partial {y}^{2}}-{k}_{c}(C-{C}_{\infty })$$

The corresponding boundary conditions for the present problem are

5 $$t < 0:u=\upsilon =0,T={T}_{\infty },C={C}_{\infty }{\rm{for}}\,\,{\rm{any}}\,x,y,$$

6 $$\begin{array}{ll}t\ge 0: & \begin{array}{l}u={u}_{w}(x,t)+{N}_{1}\nu (1+\frac{1}{\beta })\frac{\partial u}{\partial y},k\frac{\partial T}{\partial y}=-{h}_{f}({T}_{f}-T)\\ C={C}_{w}(x,t)={C}_{\infty }+{C}_{0}{x}^{m}{(1-\gamma t)}^{-2}\,{\rm{at}}\,y=0\end{array}\end{array}\},$$

7 $$u\to {u}_{e}(x,t),T\to {T}_{\infty },C\to {C}_{\infty }\,{\rm{as}}\,y\to \infty {\rm{.}}$$

where *u* and $\upsilon$ represent the velocity components in *x*− and *y*− directions respectively, *t* is time, *v* is kinematic viscosity, *σ* is the electrically conductivity, *β* is the Casson parameter, $B(x,t)={B}_{0}{x}^{(n-1)/2}{(1-\gamma t)}^{-1/2}$ is magnetic field with ${B}_{0}$ is the strength of the magnetic field, *ρ* is the fluid density, *g* is the gravitational force due to acceleration, ‘+’ sign is used for assisting flow and ‘−’ sign denotes opposing flow, *β*_*T*_ is the volumetric coefficient of thermal expansion *T* is the fluid temperature, $\alpha =\frac{k}{\rho {c}_{p}}$ is the thermal diffusivity of the fluid, *k* is the thermal conductivity, *c*_*p*_ is the specific heat at constant pressure, *q*_*r*_ is the radiative heat flux, $Q(x,t)=\frac{{Q}_{0}{x}^{n-1}}{(1-\gamma t)}$ is heat generation/absorption coefficient, *D*_*m*_ is the molecular diffusivity of species concentration, *k*_*T*_ is the thermal diffusion rate, *c*_*s*_ is the concentration susceptibility, *T*_*m*_ is the mean fluid temperature, *V*_*T*_ is the thermophoretic velocity, *k*_*c*_ is the rate of chemical reaction, ${N}_{1}(x,t)={N}_{0}{x}^{-(n-1)/2}{(1-\gamma t)}^{1/2}$ is the velocity slip factor with constant ${N}_{0}$, ${h}_{f}(x,t)={h}_{0}{x}^{(n-1)/2}{(1-\gamma t)}^{-1/2}$ is the convective heat and mass transfer with ${h}_{0}$ being constant and ${T}_{f}(x,t)={T}_{\infty }+{T}_{0}{x}^{m}{(1-\gamma t)}^{-2}$ in which ${T}_{0}$ being reference temperature and $m=2n-1$.

The expressions ${u}_{w}(x,t)$, ${u}_{e}(x,t)$, $B(x,t)$, ${T}_{f}(x,t)$, ${N}_{1}(x,t)$ and ${h}_{f}(x,t)$ are valid for $t > {\gamma }^{-1}$.

The thermophoretic velocity appears in equation (4) can be expressed as

8 $${V}_{T}=-\frac{{k}_{1}\nu }{{T}_{ref}}\frac{\partial T}{\partial y}$$

where *k*_1_ is the thermophoretic coefficient.

Now utilizing Rosseland approximation for radiation, the radiative heat flux *q*_*r*_ is given as:

9 $${q}_{r}=\frac{-4{\sigma }^{\ast }}{3{{k}_{1}}^{\ast }}\frac{\partial {T}^{4}}{\partial y},$$

where ${\sigma }^{\ast }$ is the Stefan-Boltzmann constant and ${{k}_{1}}^{\ast }$ is the mean absorption coefficient. The wall temperature excess parameter ${\theta }_{w}=\frac{{T}_{w}}{{T}_{\infty }}$. The term *T* ^4^ in equation (9) can be expressed as

10 $${T}^{4}={T}_{\infty }^{4}{[1+({\theta }_{w}-1)\theta ]}^{4}$$

Introducing suitable similarity variables

11 $$\begin{array}{rcl}\psi  & = & \sqrt{\frac{2\nu a}{(n+1)(1-\gamma t)}}{x}^{\frac{n+1}{2}}f(\eta ),\\ \eta  & = & \sqrt{\frac{(n+1)a}{2\nu (1-\gamma t)}}{x}^{\frac{n-1}{2}}y,\\ \theta  & = & \frac{T-{T}_{\infty }}{{T}_{f}-{T}_{\infty }},\\ \varphi  & = & \frac{C-{C}_{\infty }}{{C}_{s}-{C}_{\infty }},\\ {\theta }_{w} & = & \frac{{T}_{w}}{{T}_{\infty }},\\ T & = & {T}_{\infty }(1+({\theta }_{w}-1))\theta (\eta )\end{array}$$

where $\psi$ is the stream function and automatically satisfies equation (1), and given by

12 $$u=\frac{\partial \psi }{\partial y},\upsilon =-\frac{\partial \psi }{\partial x},$$

From equations (2–12), the system of equations become

13 $$\begin{array}{c}(1+\frac{1}{\beta })f\prime\prime\prime +ff^{\prime\prime} +\frac{2n}{n+1}(\varepsilon -f{^{\prime} }^{2})+\frac{2}{n+1}M(\varepsilon -f^{\prime} )+\frac{2}{n+1}(Gr\theta +Gm\varphi )\\ \quad \quad \quad \quad \,-A(\frac{1}{n+1}\eta f^{\prime\prime} +\frac{2}{n+1}f^{\prime} -\frac{2}{n+1}\varepsilon )=0,\end{array}$$

14 $$\begin{array}{c}\frac{1}{\Pr }[(1+N{(1+({\theta }_{w}-1)\theta )}^{3})\theta ^{\prime} ]^{\prime} +f\theta ^{\prime} -\frac{2(2n-1)}{n+1}f^{\prime} \theta +(1+\frac{1}{\beta })Ec{(f^{\prime\prime} )}^{2}\\ \quad \quad \,\,+(\frac{2}{n+1})MEc{(\varepsilon -f^{\prime} )}^{2}+{D}_{f}\varphi ^{\prime\prime} +\frac{2}{n+1}{\lambda }_{1}\theta -A(\frac{4}{n+1}\theta +\frac{1}{n+1}\eta \theta ^{\prime} )=0,\end{array}$$

15 $$\begin{array}{c}\frac{1}{Sc}\varphi ^{\prime\prime} +f\varphi ^{\prime} -\frac{2(2n-1)}{n+1}f^{\prime} \varphi -\tau (\varphi ^{\prime} \theta ^{\prime} +\varphi \theta ^{\prime\prime} )+Sr\theta ^{\prime\prime} -\frac{2}{n+1}R\varphi \\ \quad \quad \,-A(\frac{4}{n+1}\varphi +\frac{1}{n+1}\eta \varphi ^{\prime} )=0\end{array}$$

The dimensionless boundary conditions are

16 $$\begin{array}{rcl}f^{\prime} (\eta ) & = & 1+\delta \sqrt{\frac{n+1}{2}}(1+\frac{1}{\beta })f^{\prime\prime} (0),\\ \theta ^{\prime} (\eta ) & = & -(\frac{1}{n+1})B{i}_{1}[1-\theta (0)],\\ \varphi ^{\prime} (\eta ) & = & 1\,\,{\rm{at}}\,\,\eta =0\end{array}$$

17 $$f^{\prime} (\eta )\to \varepsilon ,\theta (\eta )\to 0,\varphi (\eta )\to 0\,{\rm{as}}\,\eta \to \infty $$

The parameters which appear in the above equations are given as

$$\begin{array}{ccc}A & = & \frac{\gamma x}{a{x}^{n}},\\ M & = & \frac{\sigma {B}_{0}^{2}}{\rho a},\\ Gr & = & \frac{g{\beta }_{T}{T}_{0}}{{a}^{2}},\\ Gm & = & \frac{g{\beta }_{C}{C}_{o}}{{a}^{2}},\\ Pr & = & \frac{\nu }{\alpha },\\ N & = & \frac{16{\sigma }^{\ast }{T}_{{\rm{\infty }}}^{3}}{3k{{k}_{1}}^{\ast }},\\ Ec & = & \frac{{u}_{w}^{2}}{{c}_{p}({T}_{f}-{T}_{{\rm{\infty }}})}\\ {\lambda }_{1} & = & \frac{{Q}_{0}}{\rho {c}_{p}a},\end{array}$$

$$\begin{array}{rcl}{D}_{f} & = & \frac{{D}_{m}{k}_{T}({C}_{w}-{C}_{\infty })}{{c}_{s}{c}_{p}({T}_{f}-{T}_{\infty })\nu },\\ Sc & = & \frac{\nu }{D},\\ Sr & = & \frac{{D}_{m}{k}_{T}({T}_{f}-{T}_{\infty })}{\nu {T}_{m}({C}_{w}-{C}_{\infty })},\\ \tau  & = & \frac{{k}_{1}({T}_{f}-{T}_{\infty })}{{T}_{erf}},\\ R & = & \frac{\nu x{k}_{c}}{{u}_{w}},\\ \delta  & = & {N}_{0}\sqrt{a\nu },\\ \varepsilon  & = & \frac{b}{a},\\ Bi & = & \frac{{h}_{0}}{k}{[\frac{2\nu }{a}]}^{1/2}\end{array}$$

where *A*, *M*, *Gr*, *Gm*, Pr, *N*, *Ec*, *λ*_1_, *D*_*f*_, *Sc*, *Sr*, *τ*, *R*, *δ*, *ε* and *Bi* are local unsteadiness parameter, magnetic parameter, thermal Grashof number, mass Grashof number, Prandtl number, radiation parameter, Eckert number, heat generation/absorption parameter, Dufour number, Schmidt number, Soret number, thermophoretic parameter, chemical reaction parameter, slip parameter, velocity ratio parameter, and Biot number.

The quantities with physical interests are the skin friction coefficient *Cf*_*x*_, local Nusselt number *Nu*_*x*_ and local Sherwood number, and are given as

18 $$C{f}_{x}{({{\rm{Re}}}_{x})}^{1/2}=\sqrt{\frac{n+1}{2}}(1+\frac{1}{\beta })f^{\prime\prime} (0)$$

19 $${({{\rm{Re}}}_{x})}^{-1/2}N{u}_{x}=-\sqrt{\frac{n+1}{2}}(1+N{(({\theta }_{w}-1)\theta (0))}^{3})\theta ^{\prime} (0)$$

20 $${({{\rm{Re}}}_{x})}^{-1/2}S{h}_{x}=-\sqrt{\frac{n+1}{2}}\varphi ^{\prime} (0)\,$$

## Results and Discussion

In order to analyze the results, numeical calculations are carried out for various values of local unsteadiness parameter *A*, Casson fluid parameter *β*, nonlinear stretching sheet parameter *n*, magnetic parameter *M*, thermal Grashof number *Gr*, mass Grashof number *Gm*, Prandtl number Pr, temperature ratio parameter *θ*_*w*_, Eckert number *Ec*, heat generation/absorption parameter *λ*_1_, Dufour number *D*_*f*_, Schmidt number *Sc*, thermophoretic number *τ*, Soret number *Sr*, chemical reaction parameter *R*, slip parameter *δ*, Biot number *Bi* and velocity ratio parameter *ε*. The comparison of results is made with the previous work of existing literature, and shown in Tables (1–4), which shows the validity of the numerical method used in this study.

**Table 1:** Comparison of $-f^{\prime\prime} (0)$ for different values of *A* with *n* = 1, *β* → ∞ and *Bi* → ∞, $M=Gr=Gm=N=\delta =\varepsilon =Ec={\lambda }_{1}={D}_{f}=Sr=\tau =R=0$.

−*f*′′(0)				
*A*	Chamkha *et al*.^14^	Sharidan *et al*.^12^	Mukhopadhyay^16^	Present results
0.8	−1.261512	−1.261042	−1.261479	−1.2610
1.2	−1.378052	−1.377722	−1.377850	−1.3777

Tables 1–3 present the values of skin friction coefficient for different values of *A*, *n* and *ε*, respectively. The present results are compared with the results of Chamkha *et al*.^14^, Sharidan *et al*.^12^, Mukhophadyay^16^, Cortell^4^, Mahapatra and Gupta^36^, Nazar *et al*.^10^, Hayat *et al*.^6^ and Mabood *et al*.^37^, and are found in excellent agreement. It is also observed from these tables that the magnitude of local skin friction coefficient increases with the increase in *n* and *ε*. Table 4 demonstrates the comparison of heat transfer rate for different values of Pr with the results of Ali^3^, Ishak and Nazar^13^ and Mabood *et al*.^37^, and revealed a good agreement. It is also found that the rate of heat transfer coefficient is higher for large values of Pr.

**Table 2:** Comparison of $-f^{\prime\prime} (0)$ for different values of *n* with, *β* → ∞, *Bi* → ∞ and $M=Gr=Gm=N=\delta =\varepsilon =Ec={\lambda }_{1}={D}_{f}=Sr=\tau =R=0$.

−*f*′′(0)			
*n*	Cortell^4^	Hayat *et al*.^6^	Present results
0.0	0.62547	0.627547	0.6276
0.2	0.766758	0.766837	0.7668
0.5	0.889477	0.889544	0.8896
1	1.0	1.0	1.0
3	1.148588	1.148593	1.1486
10	1.234875	1.234874	1.2349
100	1.276768	1.276773	1.2768

**Table 3:** Comparison of $-f^{\prime\prime} (0)$ for different values of *ε* with, *n* = 1, *β* → ∞, *Bi* → ∞ and $M=Gr=Gm=N=\delta =Ec={\lambda }_{1}={D}_{f}=Sr=\tau =R=0$.

−*f*′′(0)				
*ε*	Mahapatra and Gupta^36^	Nazar *et al*.^10^	Hayat *et al*.^6^	Present results
0.1	0.9694	0.9694	0.96938	0.9694
0.2	0.9181	0.9181	0.9181	0.9181
0.5	0.6673	0.6673	0.66732	0.6673
1	—	0.0000	0.0000	0.0000
2	−2.0175	−2.0175	−2.01750	−2.0175
3	−4.7293	−4.7296	−4.72928	−4.7294

**Table 4:** Comparison of $-\theta ^{\prime} (0)$ for different values of Pr with *β* → ∞, *Bi* → ∞ and $M=Gr=Gm=N=\delta =Ec={\lambda }_{1}={D}_{f}=Sr=\tau =R=0$.

−*θ*′(0)				
Pr	Ali^3^	Ishak *et al*.^13^	Mabood *et al*.^37^	Present results
0.72	0.8088	—	0.8088	0.8088
1	0.9691	1	1	1
3	1.9237	—	1.9237	1.9237
10	3.7207	—	3.7207	3.7207

**Figure 2:**
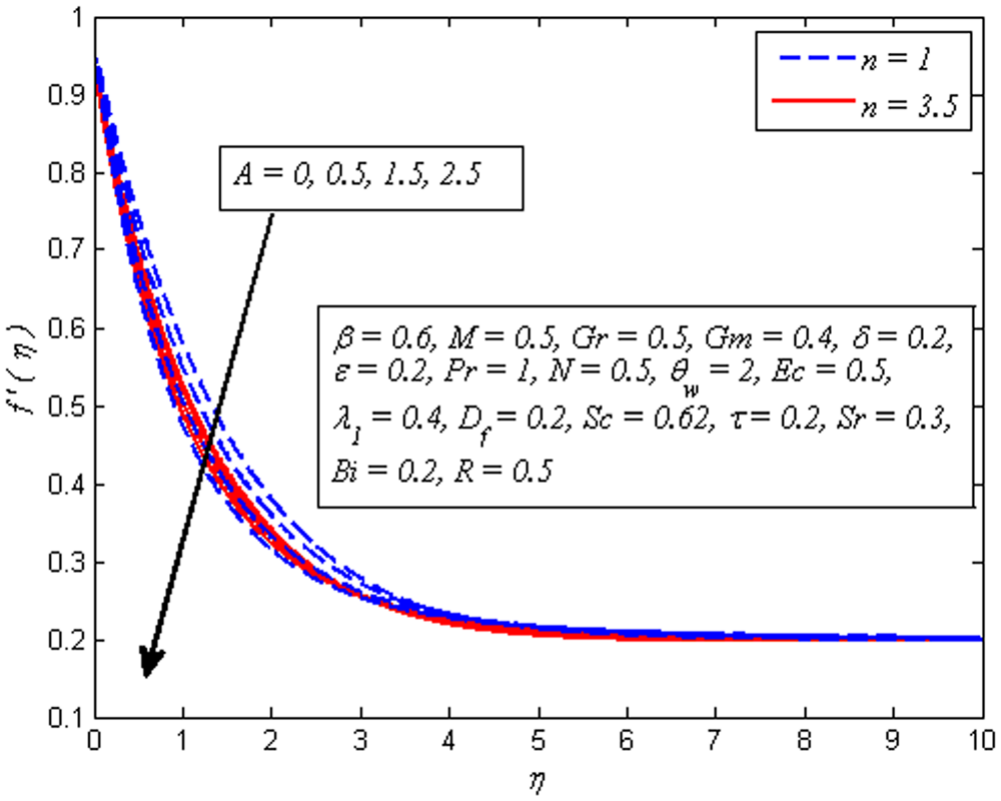
Effect of *A* on velocity for two different values of *n*.

**Figure 3:**
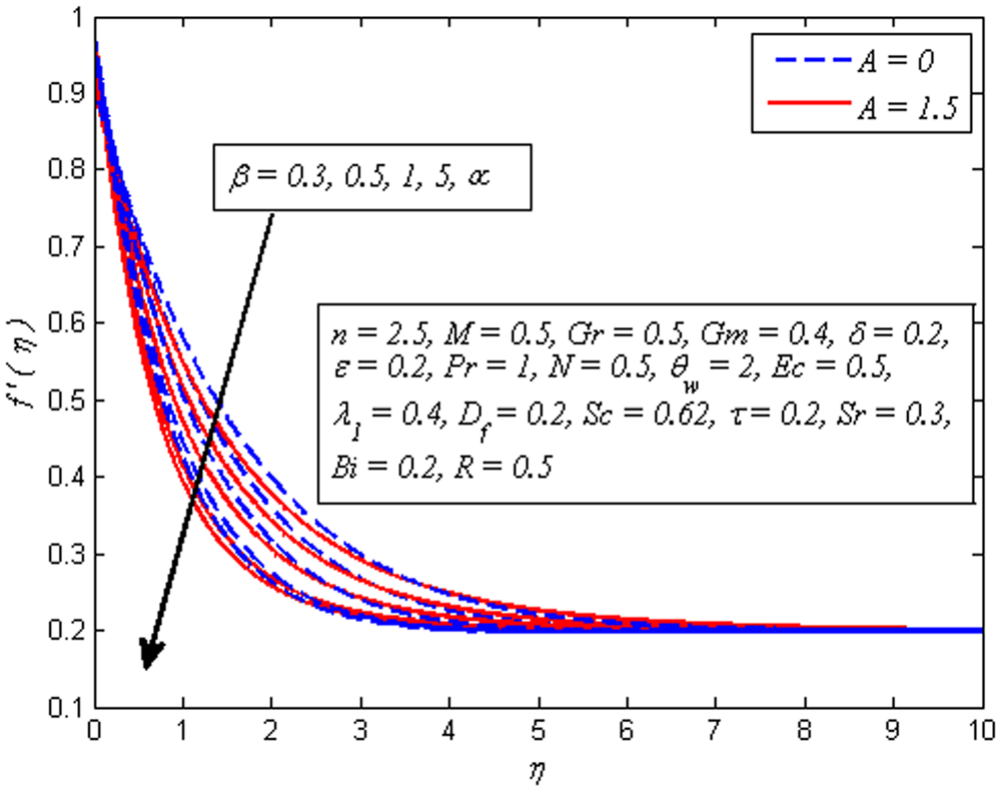
Effect of *β* on velocity for three different values of *A*.

**Figure 4:**
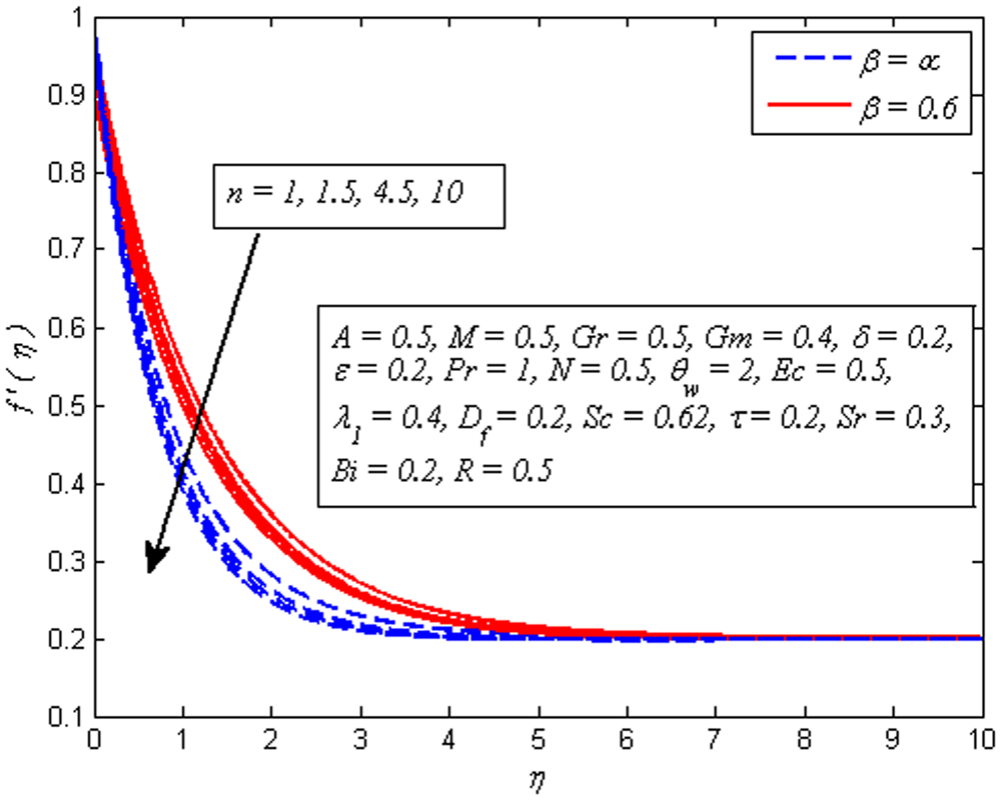
Effect of *n* on velocity for two different values of *β*.

**Figure 5:**
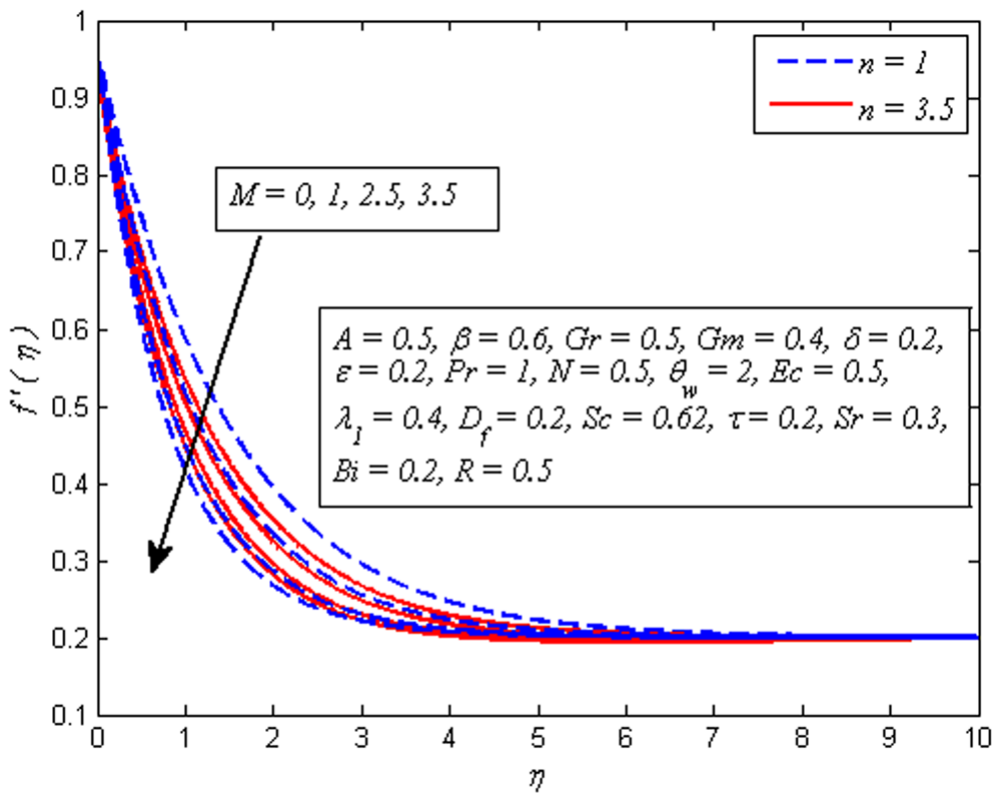
Effect of *M* on velocity for various values of *n*.

**Figure 6:**
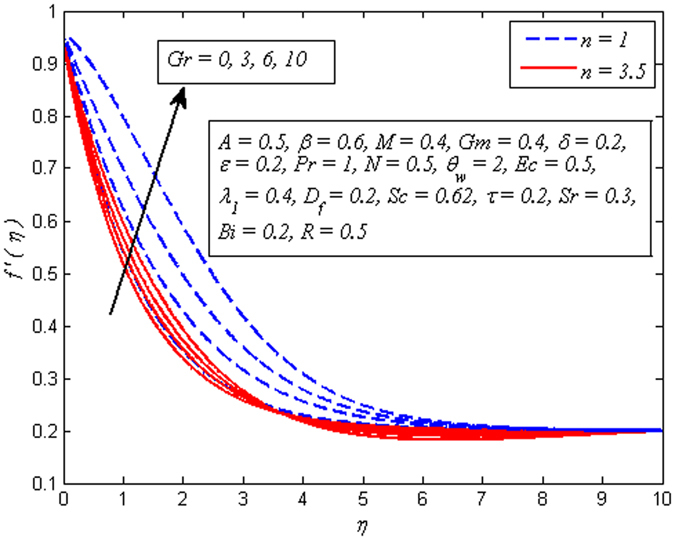
Effect of *Gr* on velocity for various values of *n*.

**Figure 7:**
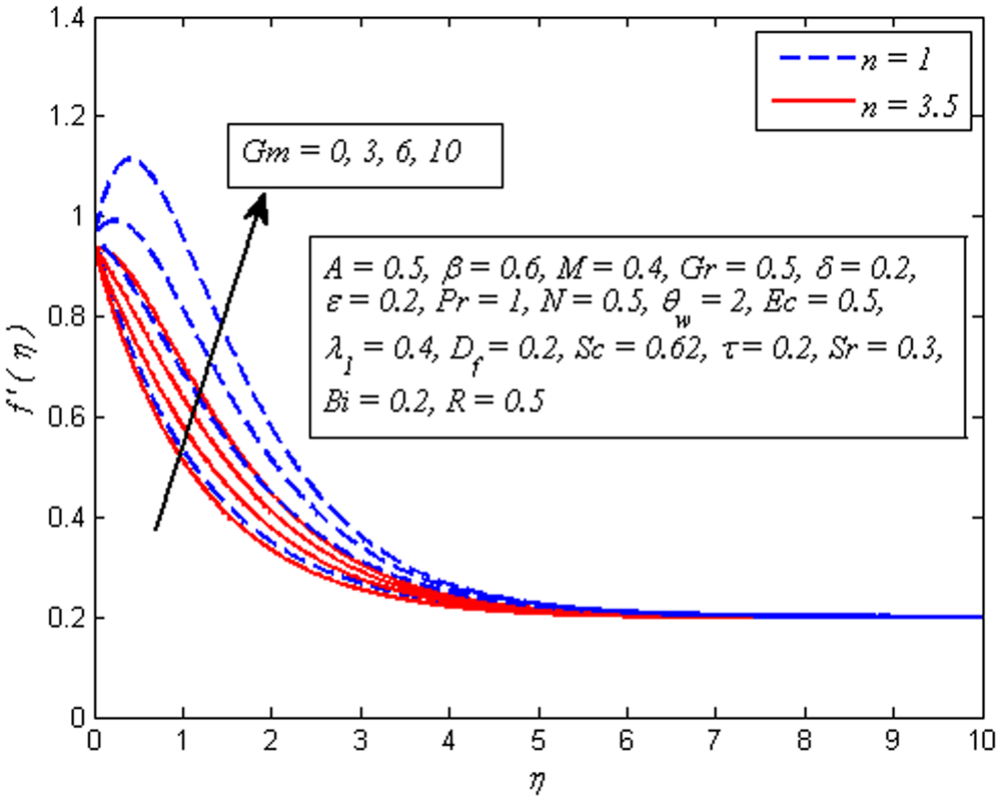
Effect of *Gm* on velocity for two different values of *n*.

**Figure 8:**
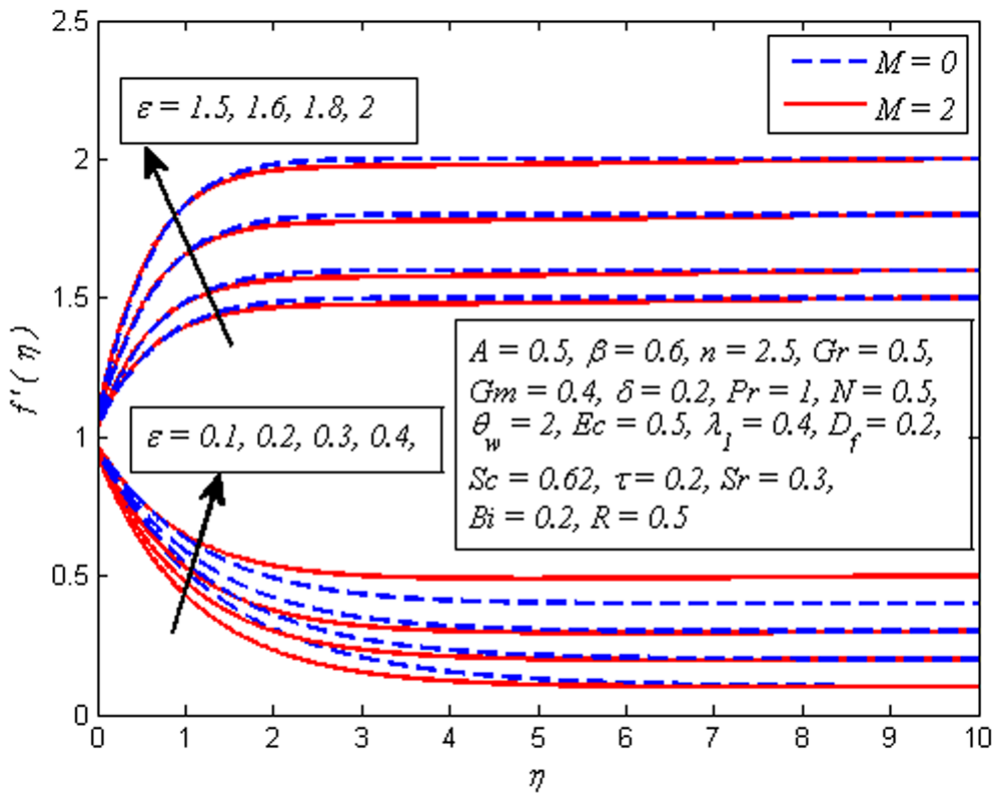
Effect of *ε* on velocity for different *M*.

**Figure 9:**
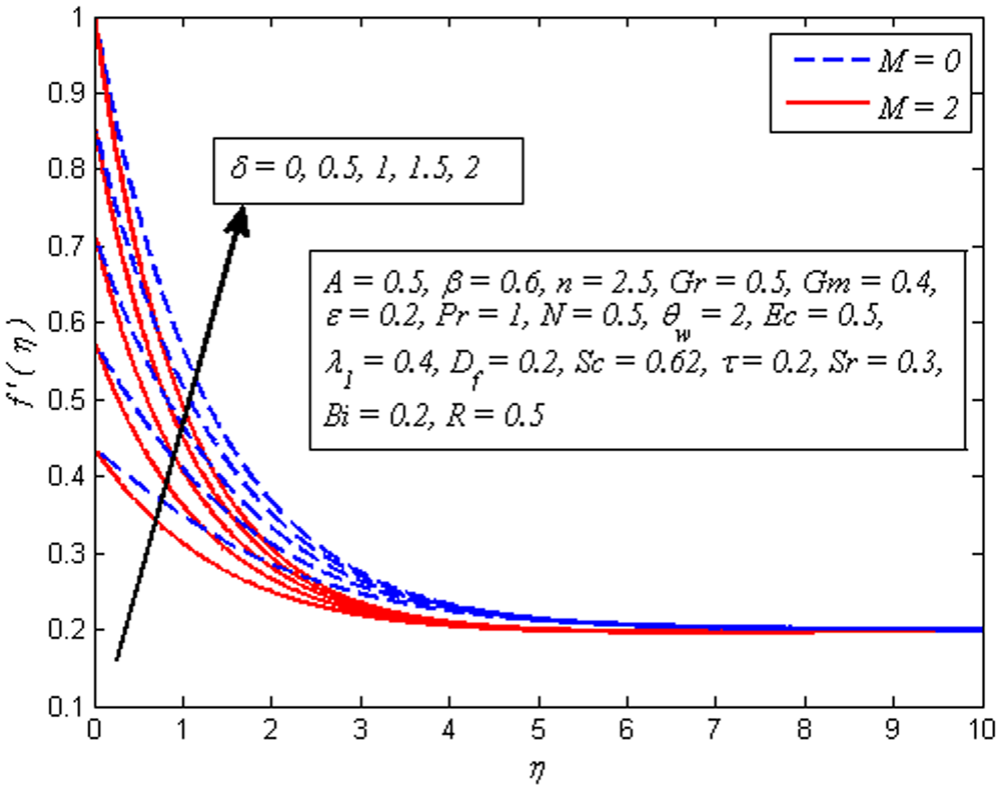
Effect of *δ* on velocity for different values of *M*.

Figures 2–9 exhibit the variation of velocity profile for various values of *A*, *β*, *n*, *M*, *Gr*, *Gm*, *δ*, and *ε*, respectively. Figure 2 reveals the effect of *A* on velocity profile for *n* = 1 and *n* ≠ 1. It is worth mentioning here that *A* = 0, represents the steady case and *A* ≠ 0 shows the unsteady case. It is noted that in both case, velocity gets lower as *A* increases. Interestingly, increase in fluid velocity for increasing values of *A* away from the sheet is also seen. However, it is more pronounced in the case of linear stretching sheet. Further, it is also observed that thickness of momentum boundary layer reduces as *A* increases. The variation of *β* on velocity profile for both steady and unsteady cases is displayed in Fig. 3. It is noteworthy here that present phenomenon reduces to Newtonian fluid when *β* → ∞. It is clear that in both cases of steady and unsteady, velocity is found to be a decreasing function of *β*. Physically, the plastic dynamic viscosity of Casson fluid increases with increases of *β* and the fluid become more viscous which results in momentum boundary layer thickness reduction. Due to low viscosity, the boundary layer of Newtonian fluid is below the Casson fluid.

The effect of *n* on velocity profile for *β* → ∞ (Newtonian fluid) and *β* = 0.6 (Casson fluid) is plotted in Fig. 4. It is seen that increasing values of *n* reduce the fluid velocity in boundary region. The momentum boundary layer thinning is also observed. Figure 5 portrays the influence of *M* on velocity profile for *n* = 1 and *n* ≠ 1. As expected increasing values of *M* lead to a decrease in the fluid velocity monotonously in the boundary region. The reason behind this phenomenon is that the resistive force known as Lorentz force produces in electrically conducting fluid, and this force has the capability to slow down the fluid flow. Figure 6 illustrates the variation of *Gr* on velocity profile for *n* = 1 and *n* ≠ 1. It is interesting to note that fluid velocity enhances with increase in *Gr* for both *n* = 1 and *n* ≠ 1. As increase in *Gr* implies a larger buoyancy force and allow more flow across the boundary. Consequently, cooling of the stretching sheet accelerates the flow and the momentum boundary layer thickness increases. A similar explanation can be given to the effect of *Gm* on the velocity profile as displayed in Fig. 7. A part from this, the influence of both *Gr* and *Gm* is more pronounced for linear stretching sheet (*n* = 1).

The influence of *ε* on velocity profile for *M* = 0 and *M* ≠ 0 is exhibited in Fig. 8. In both cases, fluid velocity is observed as increasing function of *ε*. It is interesting to note that when *ε* > 1, the flow formed boundary layer structure. It shows that increase in straining motion near the stretching sheet leads to accelerate the free stream and velocity boundary layer becomes thinner. Further, when *ε* < 1, inverted boundary layer structure is noticed. Physically, when *ε* < 1, the stretching velocity of the surface exceeds the free stream velocity.

Figure 9 depicts the influence of *δ* on velocity profile for *M* = 0 and *M* ≠ 0. It is worth mentioning here that *δ* *=* 0 represents no slip condition and *δ* ≠ 0 corresponds to velocity slip at sheet wall. Interestingly, fluid flow falls with increase of *δ* in absence as well as presence of magnetic field. A similar trend of fluid velocity is observed by Bhattacharyya *et al*.^27^. This trend is seen due to the fact that the fluid velocity near the sheet is no longer equal to velocity of stretching sheet as slip occurs at wall. In addition, the pulling of stretching sheet can be only partly transmitted to the fluid. It is also noted that velocity reduces faster in case of nonlinear stretching sheet as *δ* increases.

**Figure 10:**
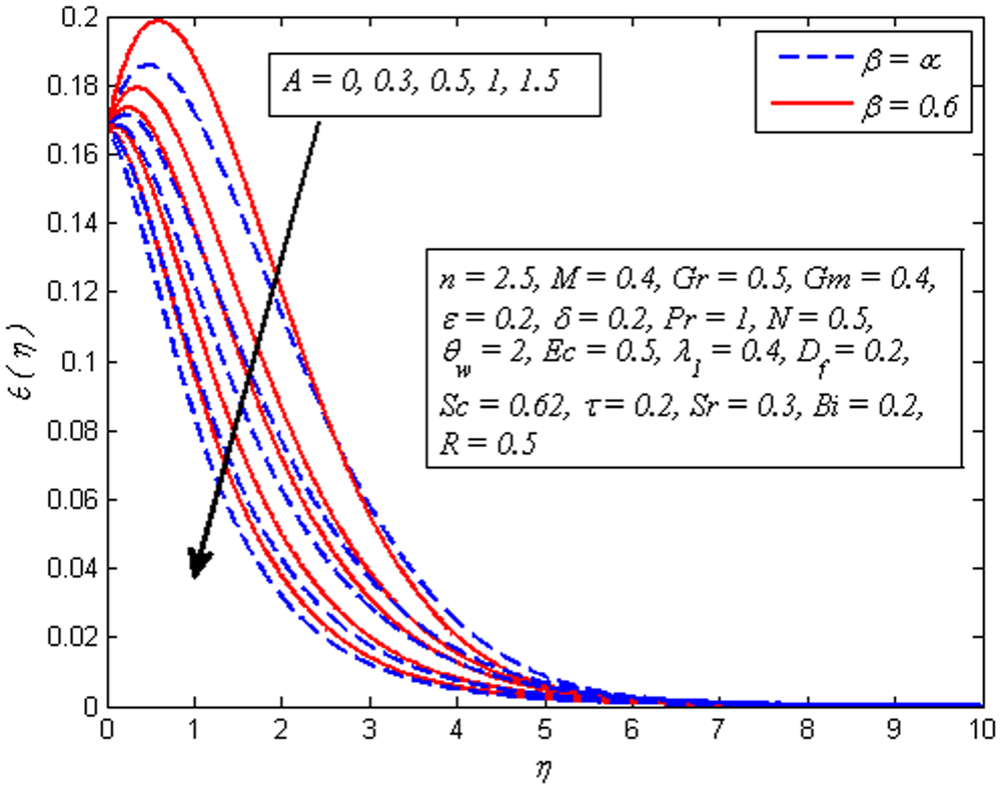
Effect of *A* on temperature for two different values of *β*.

**Figure 11:**
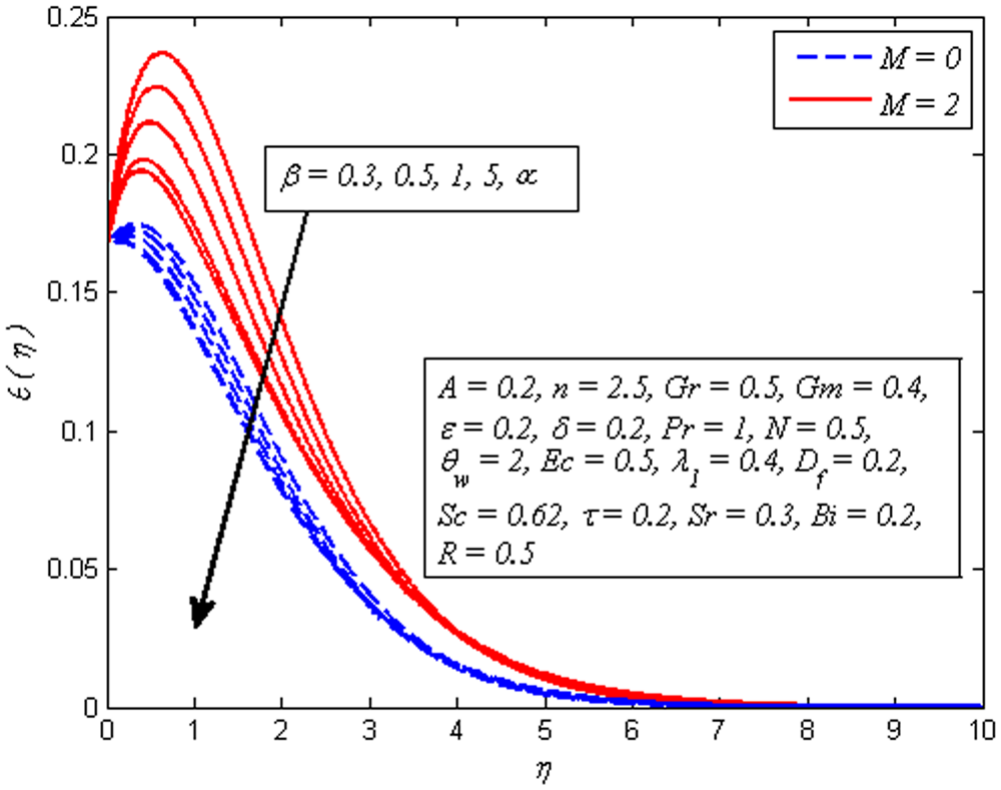
Effect of *β* on temperature for two different values of *M*.

**Figure 12:**
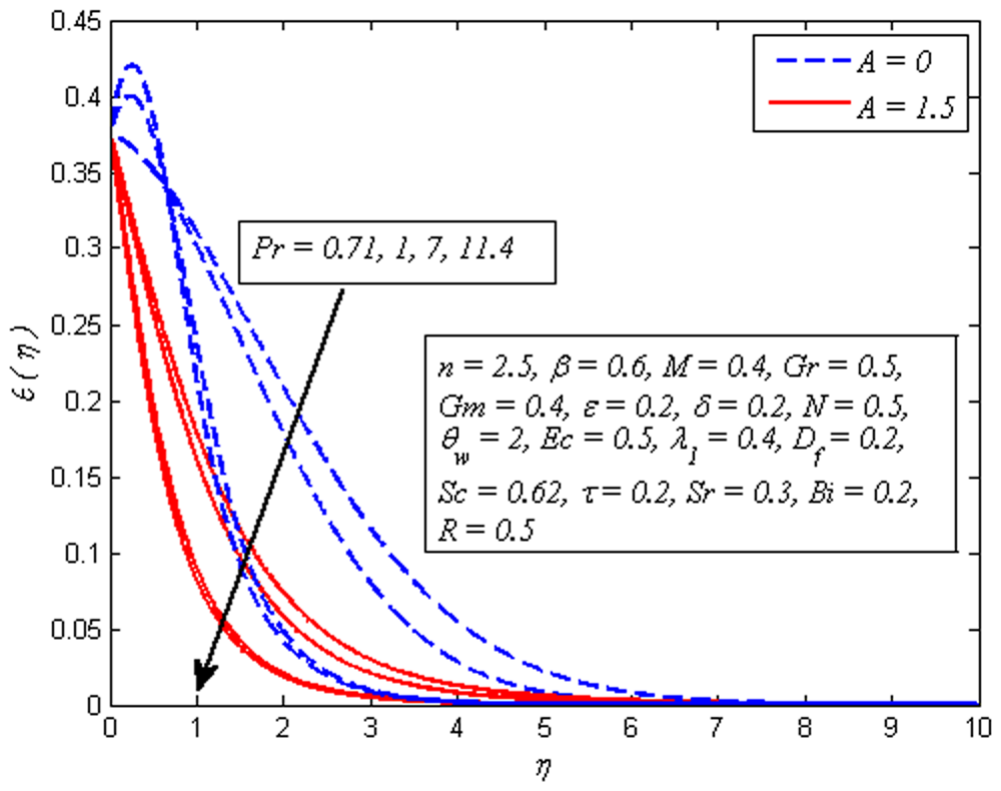
Effect of Pr on temperature for different *A*.

**Figure 13:**
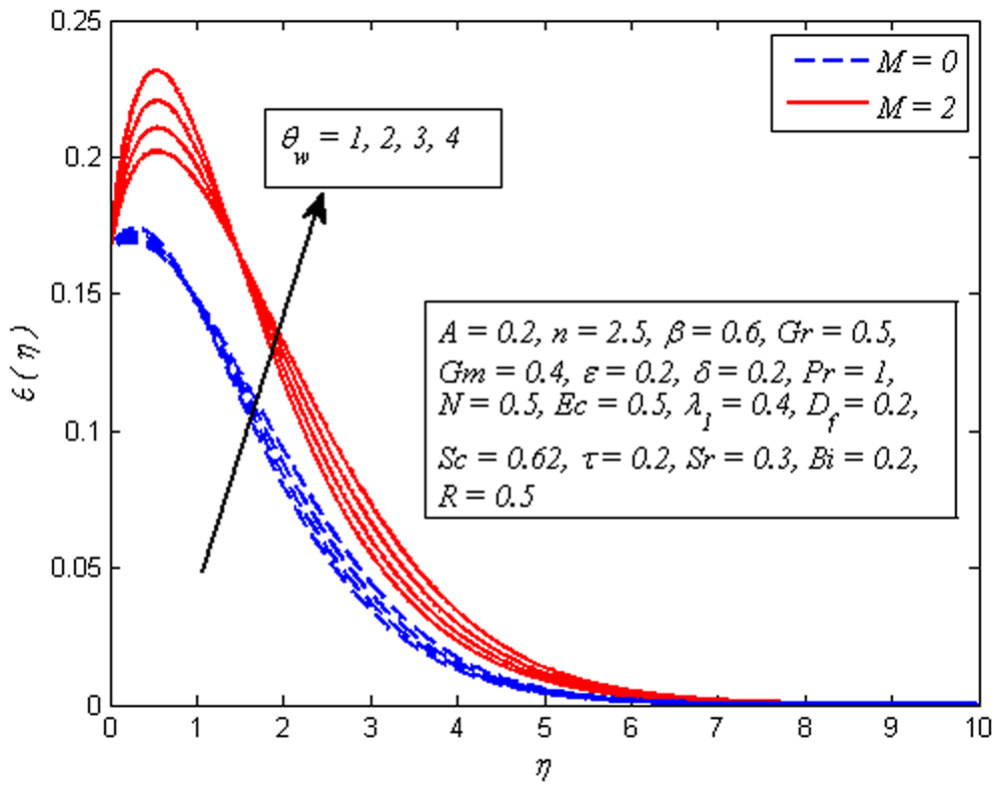
Effect of ${\theta }_{w}$ on temperature for different *M*.

**Figure 14:**
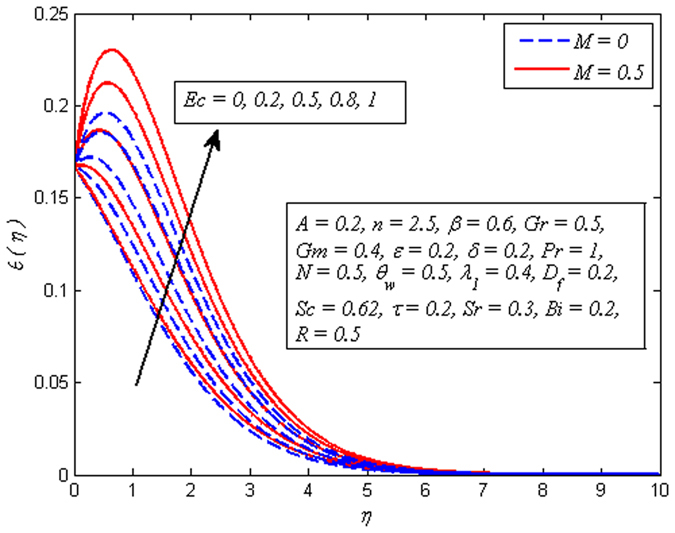
Effect of *Ec* on temperature for different *M*.

**Figure 15:**
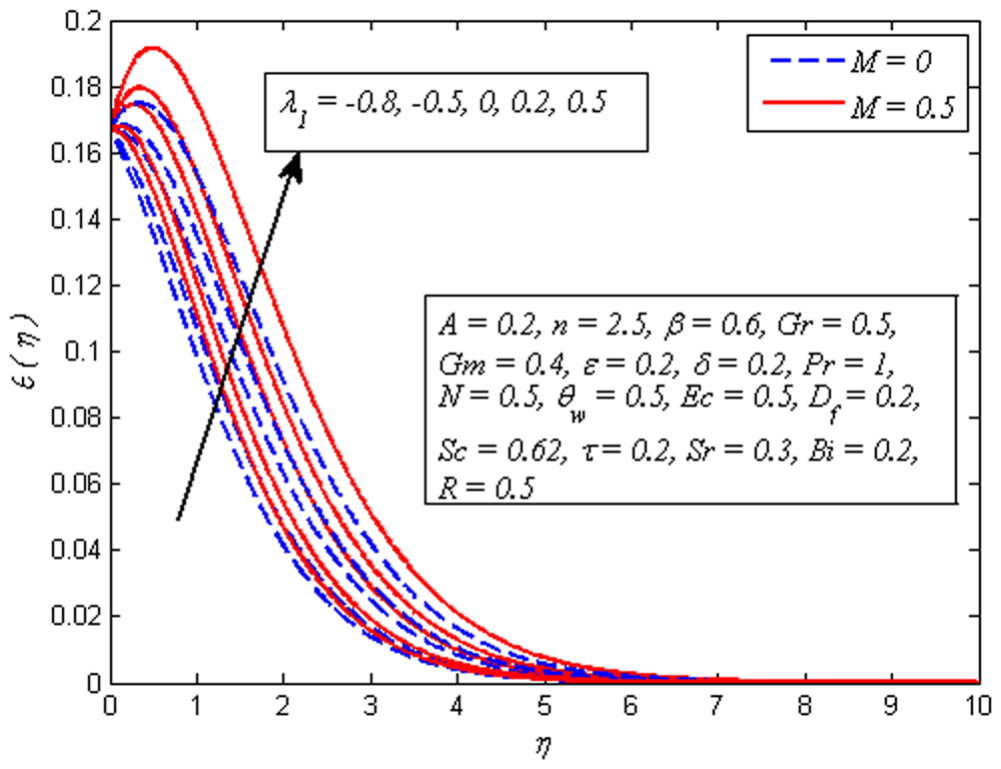
Effect of ${\lambda }_{1}$ on temperature for different *M*.

**Figure 16:**
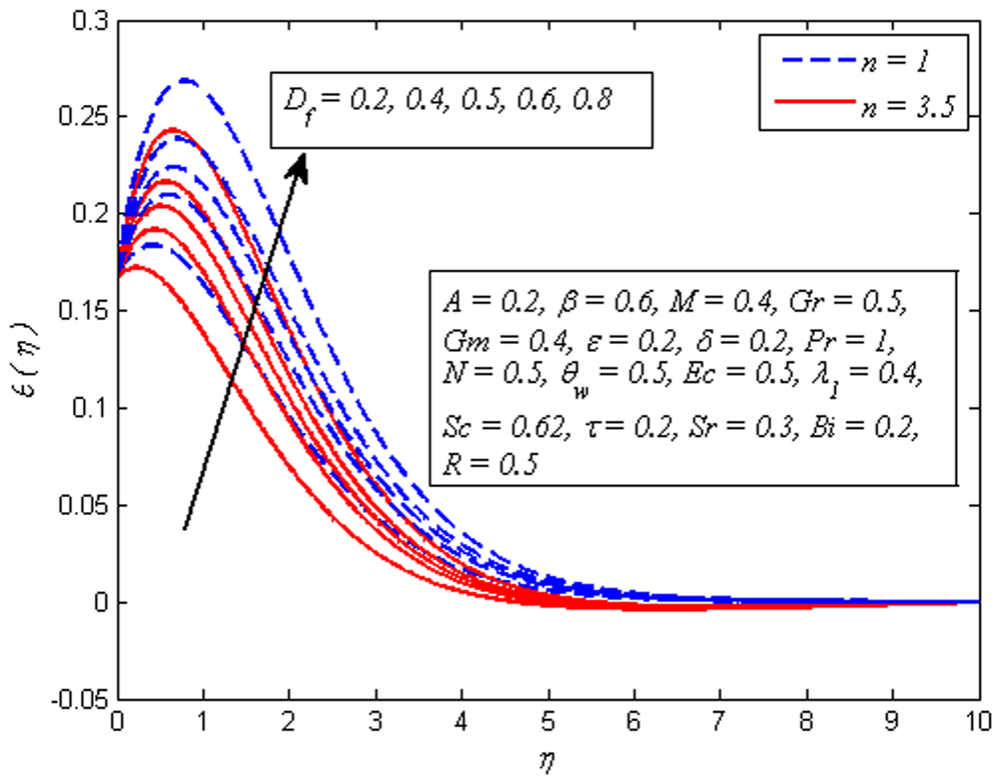
Effect of *D*_*f*_ on temperature for different *n*.

**Figure 17:**
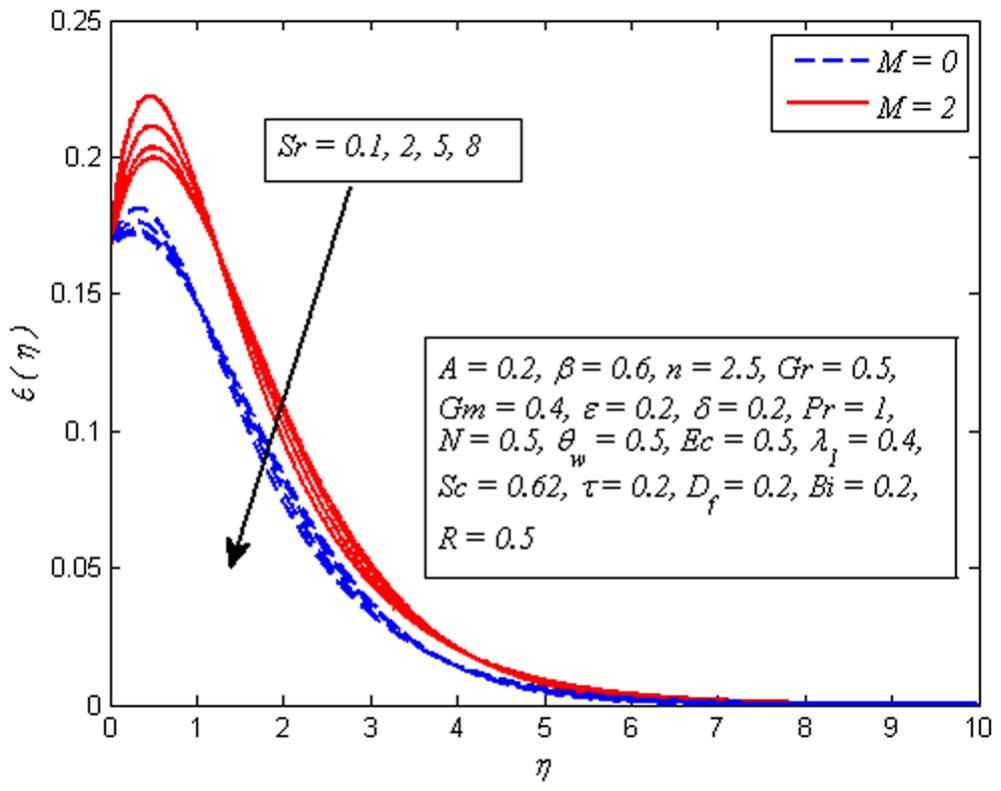
Effect of *Sr* on temperature for different *M*

**Figure 18:**
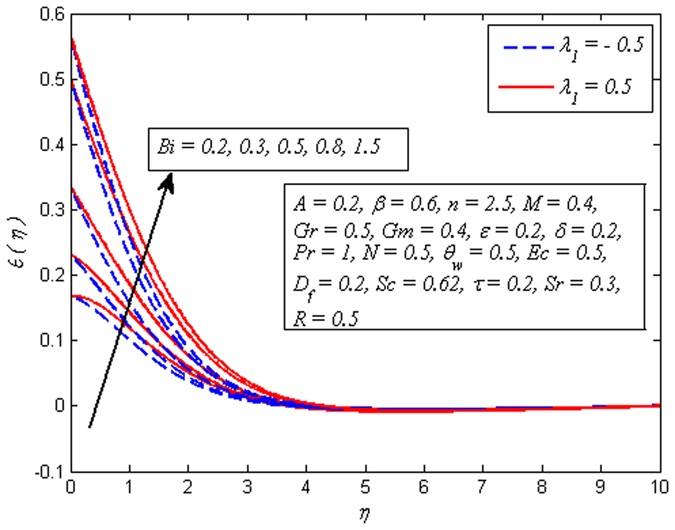
Effect of *Bi* on temperature for different *λ*_1_.

Figures 10–18 exhibit the effects of *A*, *β*, Pr, *θ*_*w*_, *Ec*, *λ*_1_, *D*_*f*_, *Sr* and *Bi* on temperature profile, respectively. It is clear from Fig. 10 that increasing values of *A* lead to reduce the temperature for both *β* → ∞ (Newtonian fluid) and *β* = 0.6 (Casson fluid). However, the dimensionless temperature gets peak values as *A* goes higher for both fluids. Figure 11 illustrates increasing values of *β* reduces the fluid temperature in both cases of *M* = 0 and *M* ≠ 0. A decrease in thermal boundary layer thickness is also observed. However, this decrease is more pronounced in the presence of magnetic field.

Figure 12 demonstrates a typical temperature profile for increasing values of Pr (i.e. Pr = 0.71,1,7,11.4 condensed air, electrolyte solution, water and water at 4 °C) for both cases of *A* = 0 and *A* ≠ 0. It is noticeable that temperature adjacent to the wall rises and then reduces with the increase in Pr. As Prandtl number increases, thickness of thermal boundary layer reduces. Physically, thermal diffusivity exceeds momentum diffusivity, i.e., heat will diffuse quickly than momentum. Temperature is observed to be squeezing closer and closer to wall as Pr increases. This implies that fluid is highly conductive when Pr ≪ 1 so that heat from sheet diffuses faster than for large Pr fluids. Therefore, in conducting flows, Prandtl number can be used to enhance cooling rate.

Figure 13 portrays that the temperature rises with the increasing values of *θ*_*w*_ in both cases of *M* = 0 and *M* ≠ 0. This is an agreement with the fact that temperature difference $({T}_{f}-{T}_{\infty })$ enhances as *θ*_*w*_ increases. Hence, thickness of thermal boundary layer rises. The variation of *Ec* on dimensional temperature profile for both *M* = 0 and *M* ≠ 0 is illustrated in Fig. 14. Clearly, higher values of *Ec* lead to higher temperature. Physically, it is realistic because dissipative heat due to viscosity and elastic deformation lead to energy storage inside the fluid region. In other words, frictional heating is the source of heat storage in the liquid. It is also observed from this figure that the temperature is more influenced for stronger values of *Ec* in the presence of magnetic field. Figure 15 demonstrates the variation of *λ*_1_ on dimensionless temperature profile for *M* = 0 and *M* ≠ 0. It is noted that temperature reduces when *λ*_1_ *<* 0, whereas *λ*_1_ > 0 enhances the fluid temperature across the boundary region. This phenomenon conforms the fact that heat absorption has a tendency to cool down the fluid temperature, whereas heat generation enhances it.

Figure 16 displays the effect of *D*_*f*_ on dimensionless temperature profile for *n* = 1 and *n* ≠ 1. It is noticed that increasing values of enhances the fluid temperature in the vicinity of boundary layer. Since *D*_*f*_ corresponds to the contribution of concentration gradient to the thermal energy flux inside the fluid. It is also seen that temperature is higher near the sheet wall as *D*_*f*_ increases. On the other hand, reverse effect is seen on temperature profile for increasing values *Sr* (see Fig. 17), i.e. temperature falls with increase in *Sr*. However, the temperature is initially higher adjacent to the wall. It is an agreement with the fact that Soret number has opposite effect to that of Dufour number. The influence of *Bi* on dimensionless temperature profile *λ*_1_ = −0.5 and *λ*_1_ = 0.5 is exhibited in Fig. 18. It would be obligatory here to mention that $Bi\to \infty$ represents a constant surface temperature case. It is noticed that temperature rises as *Bi* increases. The same effect was observed by Makinde and Aziz^30^. As Biot number is defined as the ratio of hot fluid side convection resistance to the cold fluid side convection resistance on the surface. Also, the thermal resistance of hot side fluid is proportional to *h*_*s*_, therefore increasing values of *Bi* leads to reduce the hot fluid side convection. Hence, thermal boundary layer thickness increases.

**Figure 19:**
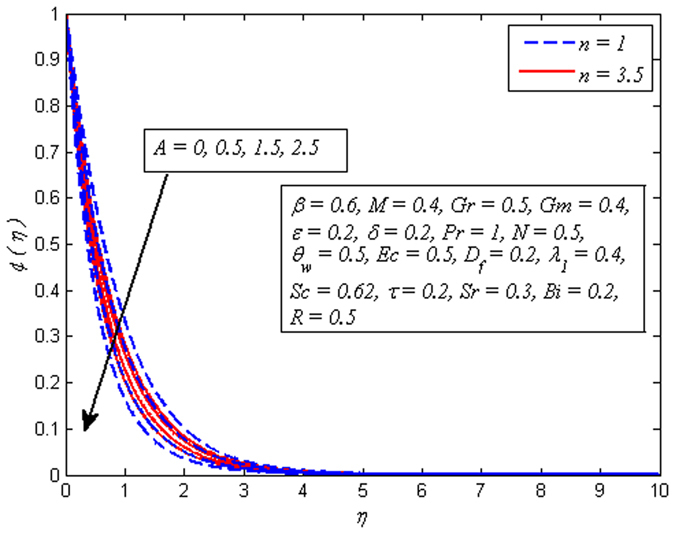
Effect of *A* on concentration for different *n*.

**Figure 20:**
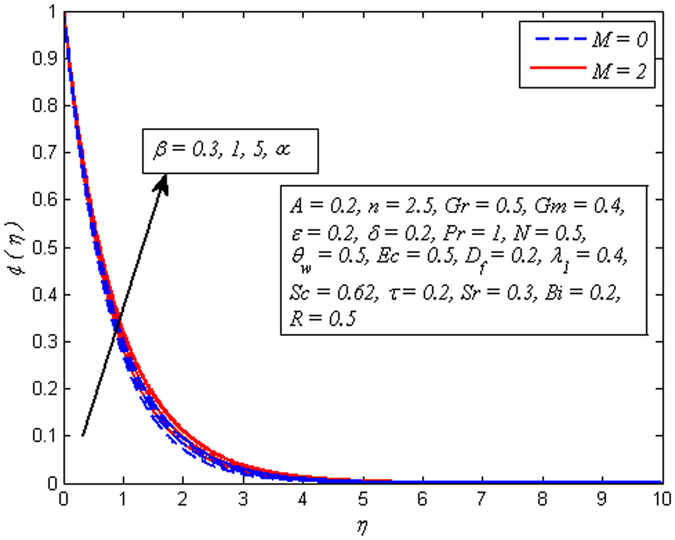
Effect of *β* on concentration for different *M*.

**Figure 21:**
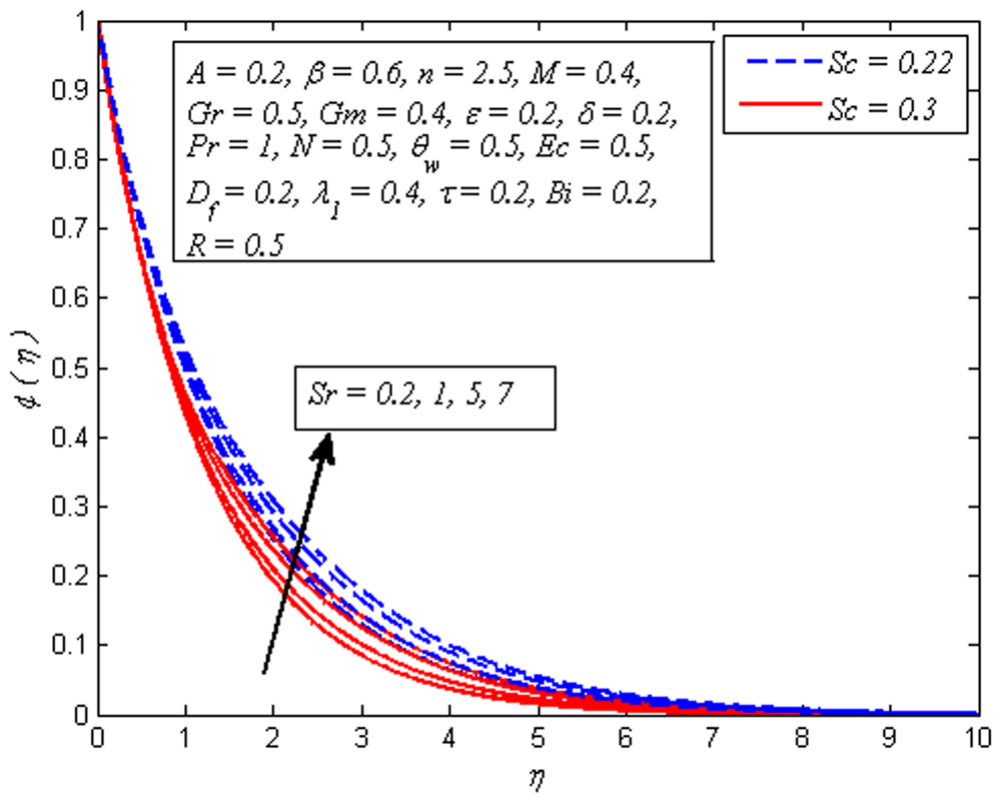
Effect of *Sr* on concentration for different *Sc*.

**Figure 22:**
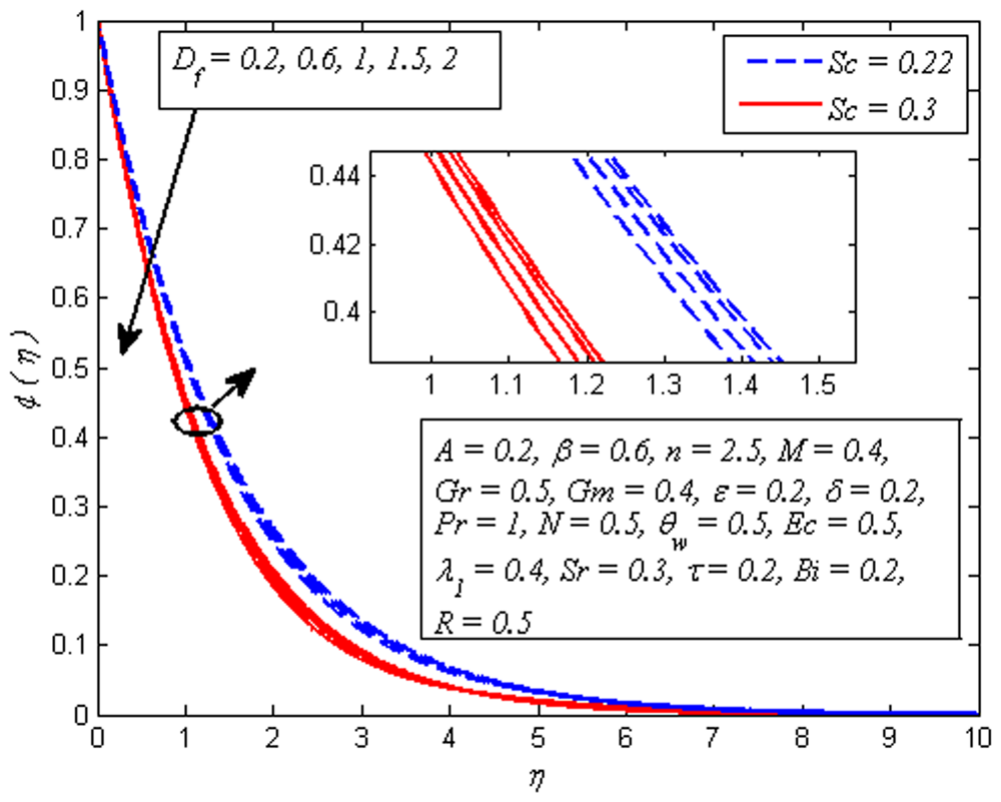
Effect of *D*_*f*_ on concentration for different *Sc*.

**Figure 23:**
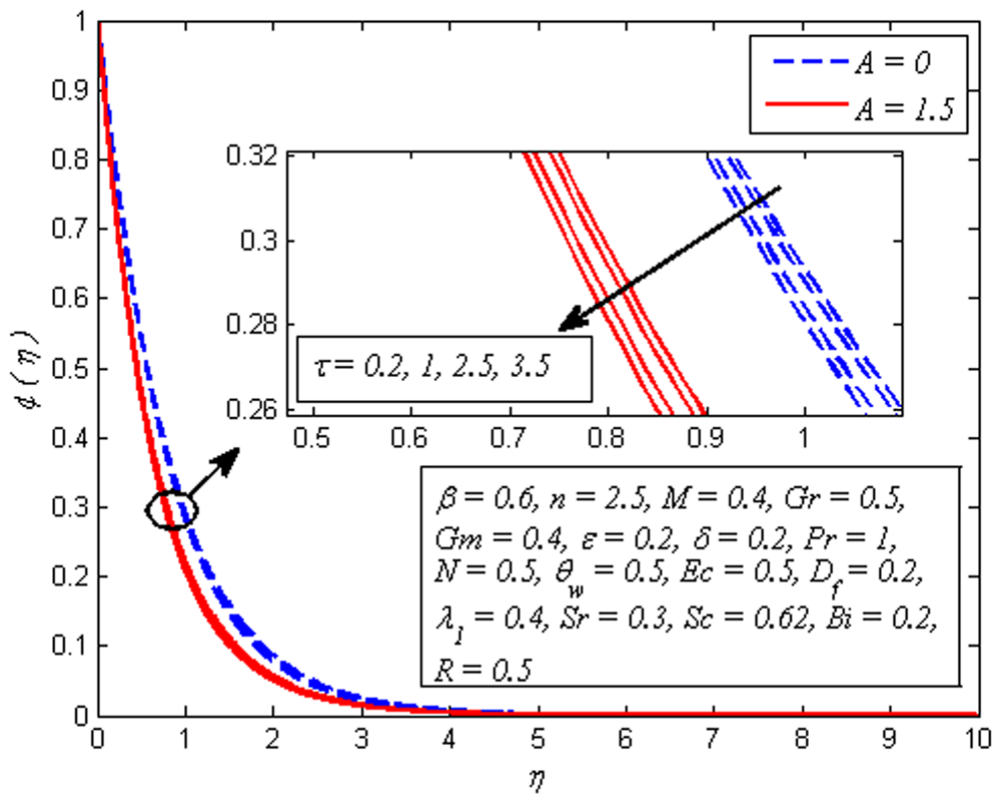
Effect of *τ* on concentration for different *A*.

**Figure 24:**
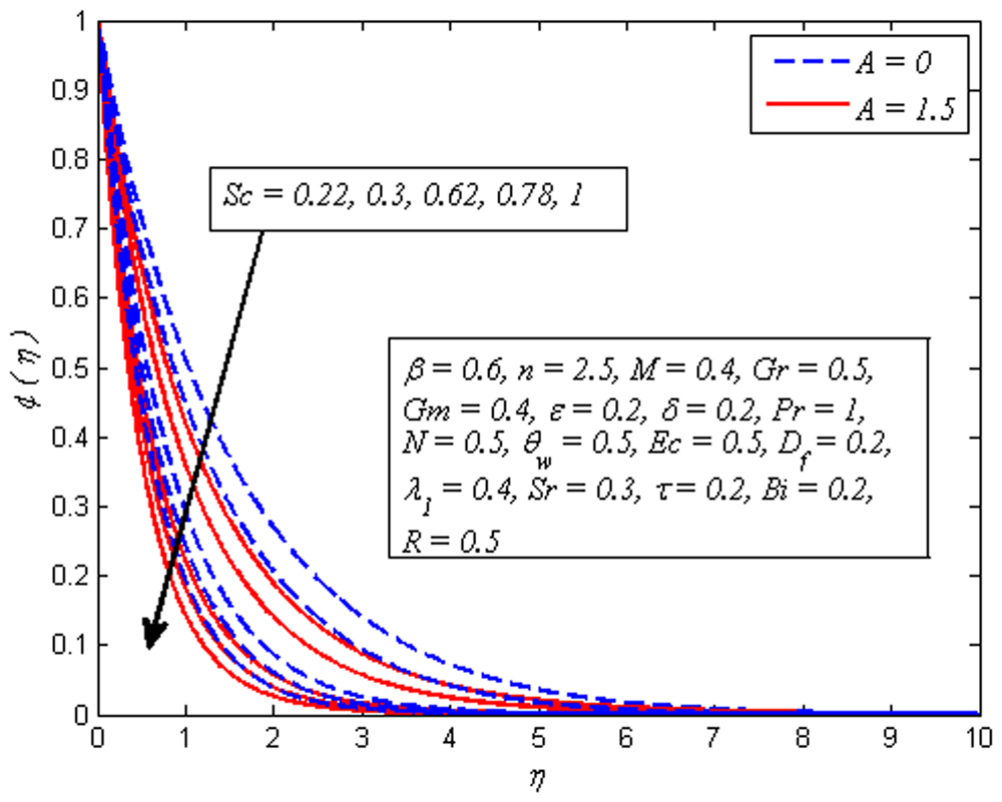
Effect of *Sc* on concentration for different *A*.

**Figure 25:**
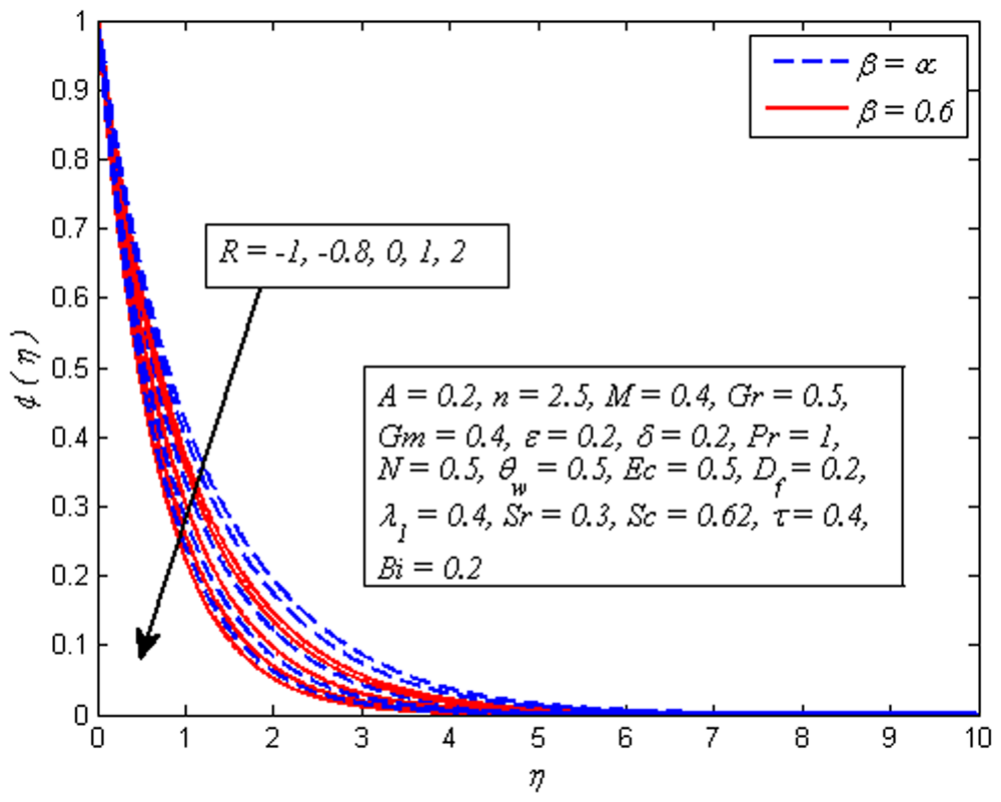
Effect of *R* on concentration for different *β*.

Figures 19–25 are plotted to insight the effects of *A*, *β*, *Sr*, *D*_*f*_, *τ*, *Sc* and *R* on concentration profile, respectively. The variation of *A* on concentration profile for *n* = 1 and *n* ≠ 1 is displayed in Fig. 19. It is noted that concentration of fluid drops as *A* increases. It can also be easily seen from this figure that the influence of *A* on concentration profile is significant in the case of nonlinear stretching sheet. Figure 20 shows the effect of *β* on concentration profile for *M* = 0 and *M* ≠ 0. It is noticed that fluid concentration is slightly enhances with increase in *β*. Like fluid velocity and temperature profiles, the influence of *β* on concentration profile is not more pronounced. The variation of *Sr* on concentration profile for two different values of *Sc* is depicted in Fig. 21. It is noticed that fluid concentration is an increasing function of *Sr*. This behavior is an agreement with the fact that Soret effect refers to mass flux from lower to higher solute concentration produced by temperature gradient. On the other hand, *D*_*f*_ has reverse effect on concentration profile, i.e. increasing values of *D*_*f*_ cause reduction in fluid concentration (see Fig. 22). It is quite obvious because the Soret and Dufour behave opposite to each other. Physically, higher values of *D*_*f*_ enhances the convection velocity due to combined effects of thermal and solutal concentration which in turn leads to increase the temperature of the fluid by lowering concentration of species.

Figure 23 demonstrates that increasing values of *τ* slightly reduces the concentration distribution. It is also observed from this figure that the concentration boundary layer become thinner as *τ* increased. Figure 24 reveals the variation of *Sc*(i.e. *Sc* = 0.22,0.3,0.62,0.78,1 represents hydrogen, helium, water vapor, Ammonia at 25 °C and CO_2_ at 25 °C, respectively) on concentration profile for *A* = 0 and *A* ≠ 0. Schmidt number Corresponds to the ratio of momentum diffusivity to species diffusivity. For *Sc* < 1, the momentum diffusivity is lesser that the species diffusivity and *Sc* > 1, momentum exceeds the species diffusivity. For *Sc* = 1, both the momentum and species diffuse at the same rate. As expected, fluid concentration reduces with increase in *Sc*. It is physically realistic due to the fact that as *Sc* is increased the concentration boundary layer will become relatively thinner than the momentum boundary layer. Figure 25 demonstrates the influence of *R* on concentration profile for *β* → ∞ (Newtonian fluid) and *β* = 0.6 (Casson fluid). For both fluids, concentration falls when *R* > 0 while rises in the case of *R* < 0. The explanation for this behavior is that destructive chemical reaction (*R* > 0) has the tendency to reduce diffusion and thereby a decrease in chemical molecular diffusivity of the species concentration. Due to which species concentration experiences retarding effect and minimize the mass transfer.

**Figure 26:**
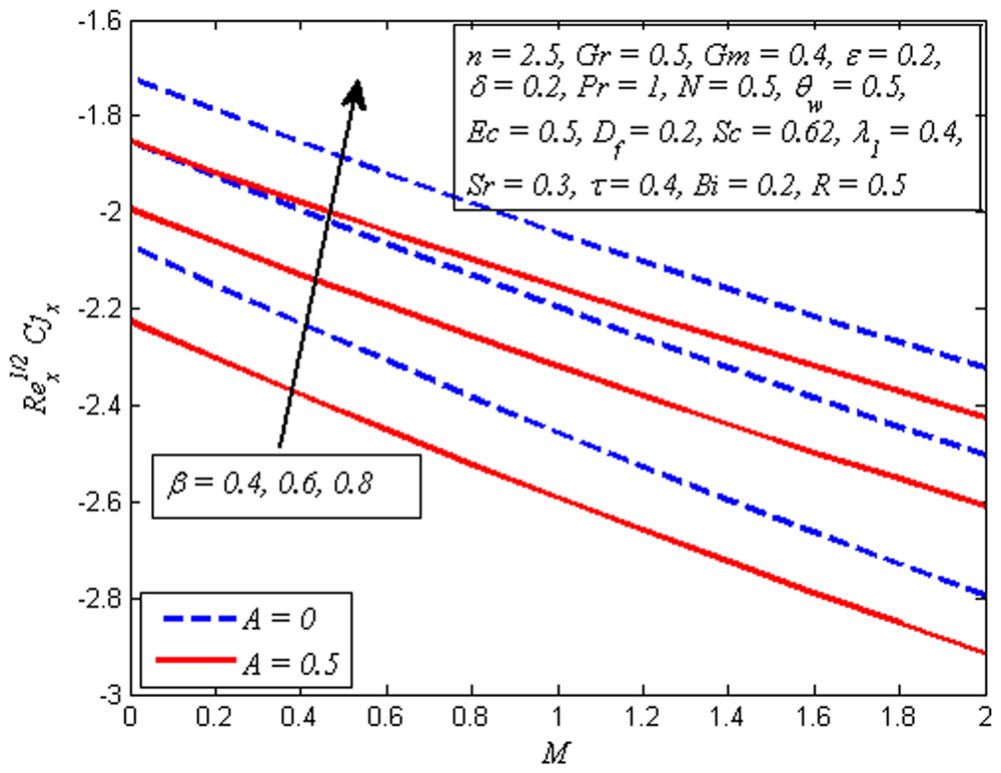
Variation of skin friction coefficient for various values of *A*, *β*, and *M*.

**Figure 27:**
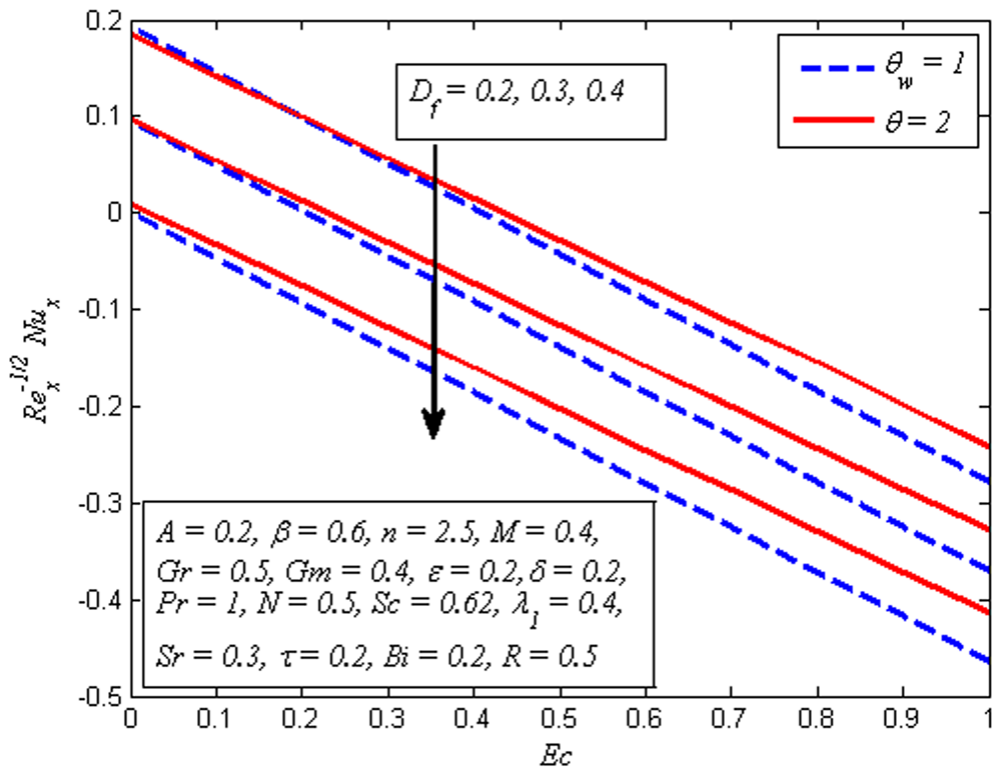
Variation of Nusselt number for various values of *D*_*f*_, ${\theta }_{w}$ and *Ec*.

**Figure 28:**
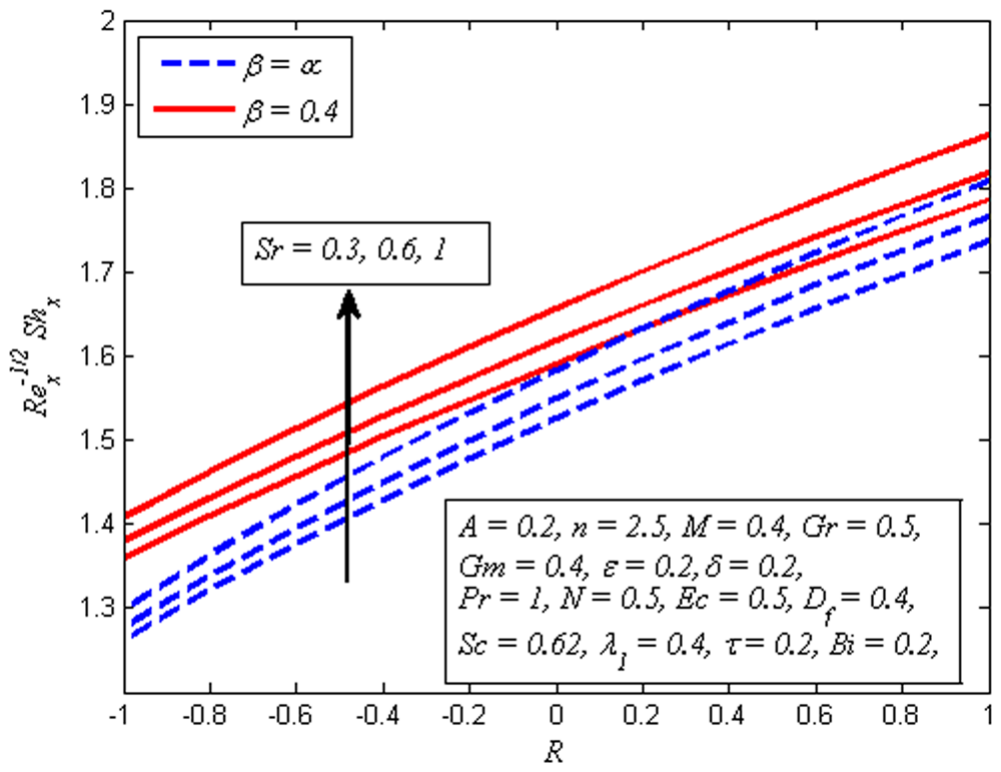
Variation of Sherwood number for various values of *β*, *Sr* and *R*.

The variation of skin friction coefficient, local Nusselt number and local Sherwood number for various values *A*, *β*, *M*, *D*_*f*_, *θ*_*w*_, *Ec*, *R* and *Sr* are displayed in Figs 26–28, respectively. It is worth mentioning that the skin friction coefficient $({{\mathrm{Re}}_{x}}^{1/2}C{f}_{x})$ is negative for all values of *A*, *β*, and *M*. Physically, this show that surface of stretching sheet applies a drag force on fluid and opposite to this for positive values.

The behavior of Nusselt number for increasing values of *D*_*f*_, *θ*_*w*_ and *Ec* are shown in Fig. 27. It is seen that negative wall slope of temperature gradient is a decreasing function of *D*_*f*_, *θ*_*w*_ and *Ec*. Finally, Fig. 28 illustrates the variation of Sherwood number for various values of *β*, *Sr* and *R*. It is noticed that mass transfer rate declines with increase in *β* while reverse effect is observed for increasing values of *Sr* and *R*.

## Conclusions

The influence of Soret and Dufour on unsteady mixed convection flow of Casson fluid over a nonlinearly stretching sheet under the influence of viscous dissipation and heat generation/absorption was investigated numerically. The governing nonlinear partial differential equations are transformed into nonlinear ordinary differential equations by employing similarity transformations. The resulting equations were solved numerically by an implicit finite difference scheme. In order to check the accuracy and validate the present method, results are compared with the results of existing literature. An excellent agreement is noticed with those results. Influence of local unsteadiness parameter *A*, Casson fluid parameter *β*, nonlinear stretching sheet parameter *n*, magnetic parameter *M*, thermal Grashof number *Gr*, mass Grashof number *Gm*, Prandtl number Pr, temperature ratio parameter ${\theta }_{w}$, Eckert number *Ec*, heat generation/absorption parameter ${\lambda }_{1},$ Dufour number *D*_*f*_, Schmidt number *Sc*, thermophoretic number *τ*, Soret number *Sr*, chemical reaction parameter *R*, slip parameter *δ*, Biot number *Bi* and velocity ratio parameter *ε* on velocity, temperature and concentration profiles as well as wall shear stress, heat and mass transfer rates are displayed graphically and discussed. Some of the interesting findings of this study are summarized as:

(i) An increase in *A* results a decrease in velocity gradient whereas it raises heat transfer rate.
(ii) The magnitude of wall shear stress and mass transfer rate risen with *β* where opposite trend was noticed in heat transfer rate.
(iii) The fluid velocity is significantly increased with *Gr* and *Gm* when *n* = 1.
(iv) The effect of *Ec* on dimensionless temperature is more significant in the presence of magnetic field.
(v) The fluid temperature is higher for the MHD flow when ${\theta }_{w} > 1$.
(vi) The influence of *D*_*f*_ on temperature is more pronounced as compared to *Sr*.
(vii) The fluid concentration is less influenced with *τ*.
(viii) The mass transfer rate is higher for higher values of *Sr*.As part of reference editing, missing details have been added, where required, from Pubmed or other relevant sites. Please confirm the correctness of the added information.I confirmed the added information.
